# Correction: DeepGANnel: Synthesis of fully annotated single molecule patch-clamp data using generative adversarial networks

**DOI:** 10.1371/journal.pone.0325021

**Published:** 2025-05-20

**Authors:** Sam T. M. Ball, Numan Celik, Elaheh Sayari, Lina Abdul Kadir, Fiona O’Brien, Richard Barrett-Jolley

The lower sub-panel image of Fig 5D is incorrect in the published article [1]. Please see the correct Fig 5 here.

**Fig 5:**
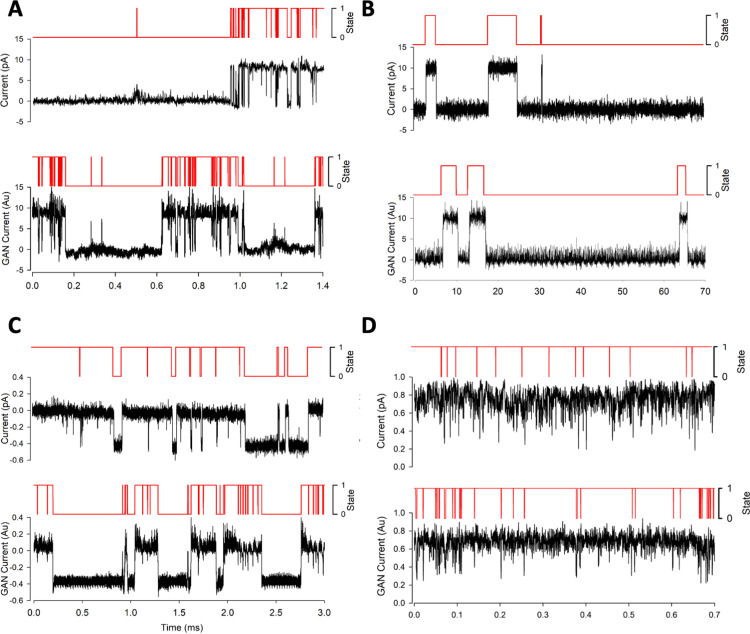
Sample data from both real dataset and DeepGANnel model output from numerous ion channels. In each subpanel, real raw and labelled data is displayed on top with simulated data below. (A): “Channel phenotype A” canine articular chondrocyte sample data and model output. (B): “Channel phenotype B”, WinEDR simulated sample data and model output. (C): “Channel phenotype C” tracheal chondrocyte sample data and model output. (D): “Channel phenotype D” PVN sample data and model output.

## References

[1] Ball STM, Celik N, Sayari E, Abdul Kadir L, O’Brien F, Barrett-Jolley R. DeepGANnel: Synthesis of fully annotated single molecule patch-clamp data using generative adversarial networks. PLoS One. 2022;17(5):e0267452. doi: 10.1371/journal.pone.0267452 35536793 PMC9089889

