# Supplementary material for: DeepGANnel: Synthesis of fully annotated single molecule patch-clamp data using generative adversarial networks
Source: PLoS One. 2022 May 10;17(5):e0267452. doi: 10.1371/journal.pone.0267452 (PMC9089889; doi:10.1371/journal.pone.0267452)
Supplement: S1 Appendix — (DOCX) [file pone.0267452.s001.docx]

**Appendix 1**

Our neural networks are constructed by using so called "layers" of types of neruons. In this appendix we will go into substantially more detail as to how these "layers" work, and how the networks operate together during training. The full model architecture of both generator and discriminator including shapes are included in Appendix 2 and 3 respectively.

***Dense Layer***

Dense layers, also known as "fully connected" layers, have each neuron connected to every neuron or data point of the previous layer (Fig 9). Let $x=(x_{1},x_{2},\ldots x_{n})$ be the inputs to the layer (either from the latent noise vector for the generator or the penultimate layer output for the discriminator). Then the output vector $y$ of size $m$ is given by:

$$y=a\left( W\cdot x+b \right)$$

Where $W$ is the $\left( n\times m \right)$ matrix of weights that are adjusted throughout the training process by our optimiser funciton, $b$ is the bias term, and $a$ the activation function, which for all layers in both of our models is the Leaky Rectified Linear Unit (Leaky ReLU), that operates elementwise on the vector input by the following mechnaism:

$$f\left( x \right)=\left\{ \begin{aligned} \alpha x, &x<0 \\ x, &x\geq0 \end{aligned} \right.$$

For all our functions, $\alpha$ is taken to be 0.3.

**
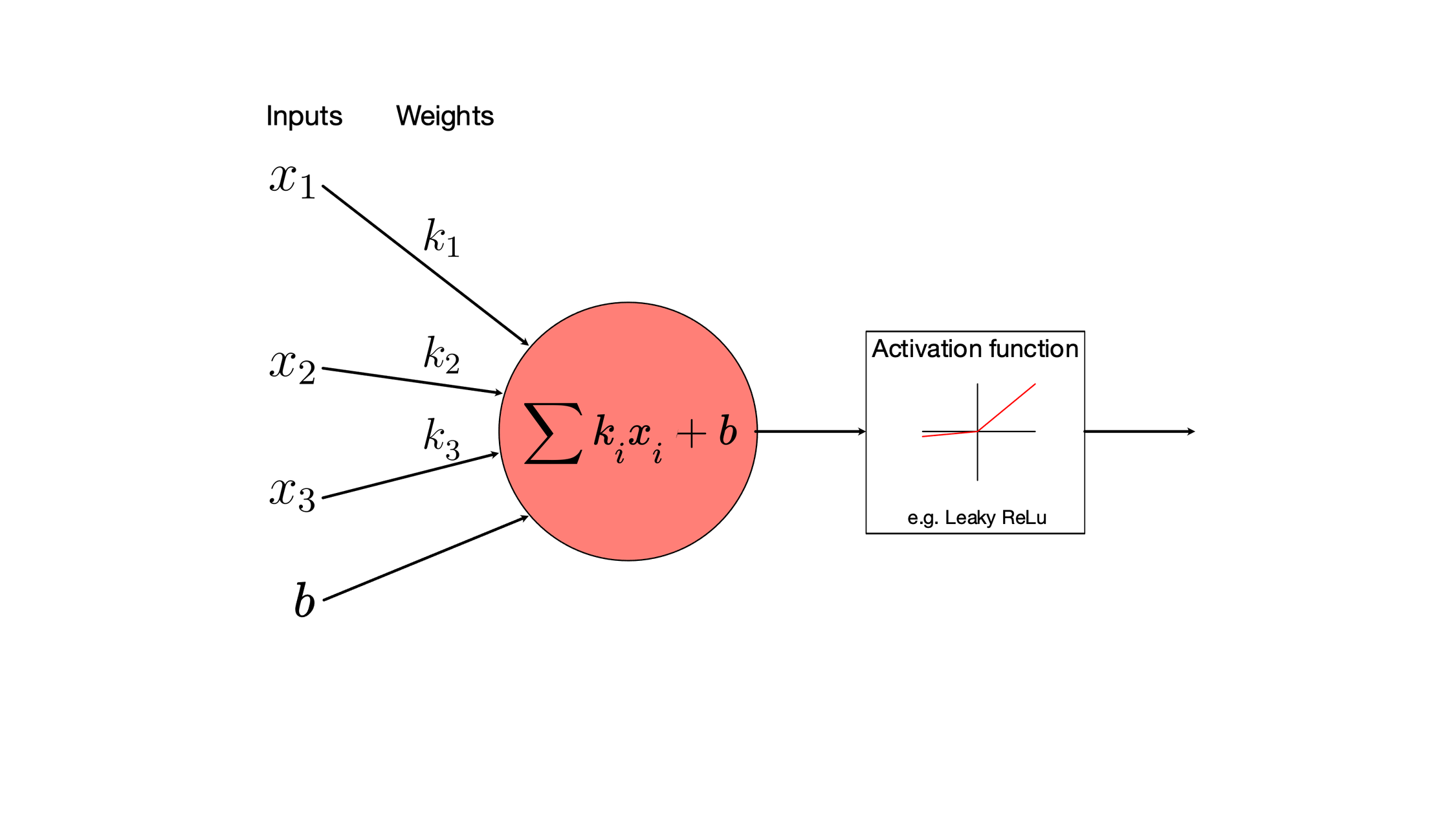
**

**Fig. 9. Diagram of how a simple neuron operates in a neural network.**

*A number of inputs (either from an input vector or a series of outputs from other neurons) are multiplied by a series of trainable parameters. These are then totalled along with a non-trainable bias term and passed through to an activation function for output. The main disadvantage of using layers of simple neurons that have inputs from every previous output is parameter cost. As more neurons are added the number of parameters exponentially increases leading to poor training performance and the danger of overfitting.*

***Convolutional and Deconvolutional Layers***

Convolutional and deconvolutional layers work via very similar means to achieve down-sampling and up-sampling respectively. Typically two dimensional layers are used for image analysis, but the principles can be extended to any number of dimensions.

At its core, the process involves using a kernel $K$ of a chosen size with trainable parameters that passes over the input and is multiplied by a sub-matrix of the input $X$ - the size of this submatrix, and the order of operations dictates whether downsampling or upsampling occurs.

In downsampling, each element $y_{i,j}$ of the output matrix $Y$ is given as follows - for simplicity here the kernel size is $\left( 3\times3 \right)$ and the stride is 1. Fig 10 shows visualisations of the areas chosen and the mappings. they correspond to.

$$y_{i,j}= b_{i,j}+\sum_{c=1}^{3} \sum_{d=1}^{3} K_{c,d}\cdot X_{(i+c-1),(j+d-1)}$$

The dimensions of the kernel are adjusted by changing the bounds of the sums, and the indexes of the input matrix (the $-1$ term becomes $-\left\lfloor s/2 \right\rfloor$, where $s$ is the desired kernel size). A stride can be applied in either or both directions by incrementing $i$ and/or $j$ in values other than one, and simply ignoring the resulting "gaps" in the output matrix. Typically when either of the indexes of $X$ are outside of the bounds of $X$ (for example, when the index is negative), the value is taken as zero (this is refered to as padding).


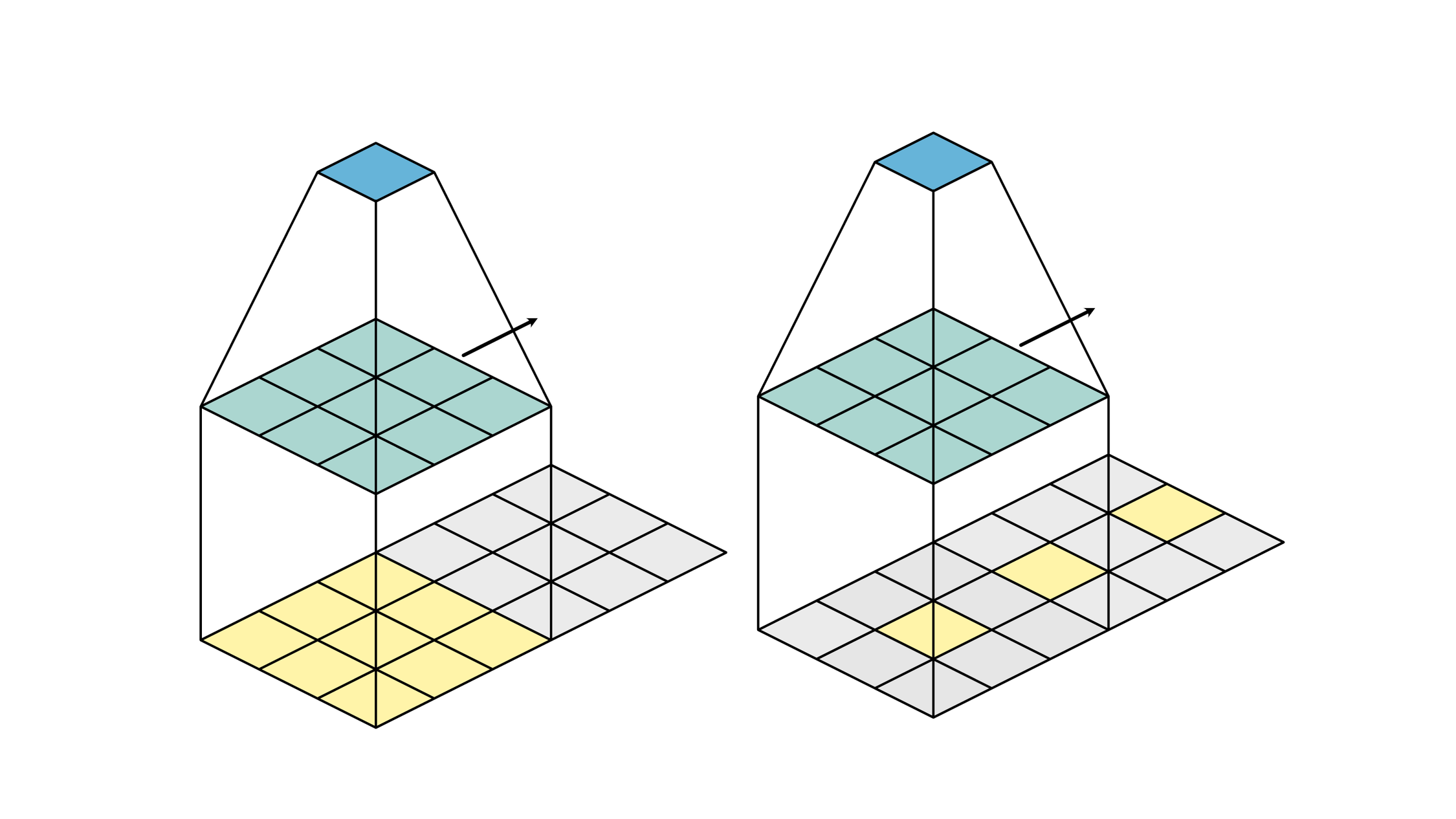


**Fig. 10. Diagram of Convolutional and deconvolutional layers.**

*Typically used in image analysis, convolutional (left) and deconvolutional layers (right) work by passing a series of trainable kernels (green) over an image, taking the sum of the pairwise product of the kernel and each submatrix of the input. As these kernels “scan” across the image, features such as edges are detected, and “feature maps” are built as the network increases in depth. By adding zero padding between each input, the downscaling effect of the scanning process becomes upscaling, which can be used in generation instead of detection.*

***Max Pooling Layers***

Max pooling layers achieve down sampling but are far simpler than convolutional layers. A set of $n\times n$ submatricies are taken, and for each one the maximal value of each submatrix is passed to a new, smaller submatrix. For example, when $n=2$, the output matrix will be of size $\frac{n}{2}\times\frac{n}{2}$. This quickly and cheaply achieves downsampling but does not recognise patterns as strongly as convolutional layers.

***Dropout Layers***

Droupout layers are a regularisation technique designed to stop a model overfitting. They work by disabling a randomly selected proportion of the inputs, with the proportion being defined with a probability hyperparameter $p$.

***Batch Normalisation***

Batch normalisation is another regularisation technique. In theory, a layer's parameters are adjusted based on the assumption that the previous layer's parameters are static; however this is clearly not the case. A batch normalisation layer scales the output of a layer to have a mean of 0 and standard deviation of 1 for each batch to realign the data with this assumption.
