# Supplementary figures and images for: DeepGANnel: Synthesis of fully annotated single molecule patch-clamp data using generative adversarial networks
Source: PLoS One. 2022 May 10;17(5):e0267452. doi: 10.1371/journal.pone.0267452 (PMC9089889; doi:10.1371/journal.pone.0267452)

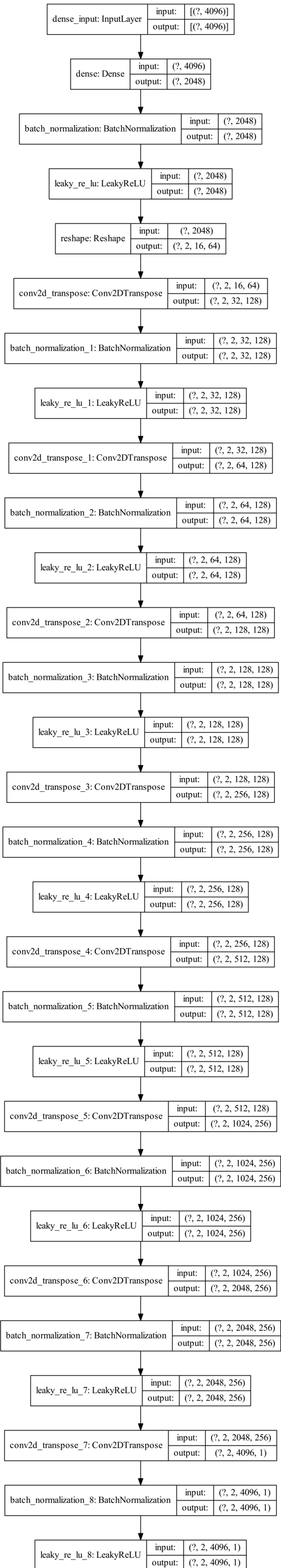

Supplement: S2 Appendix — (PDF) [file pone.0267452.s002.pdf]

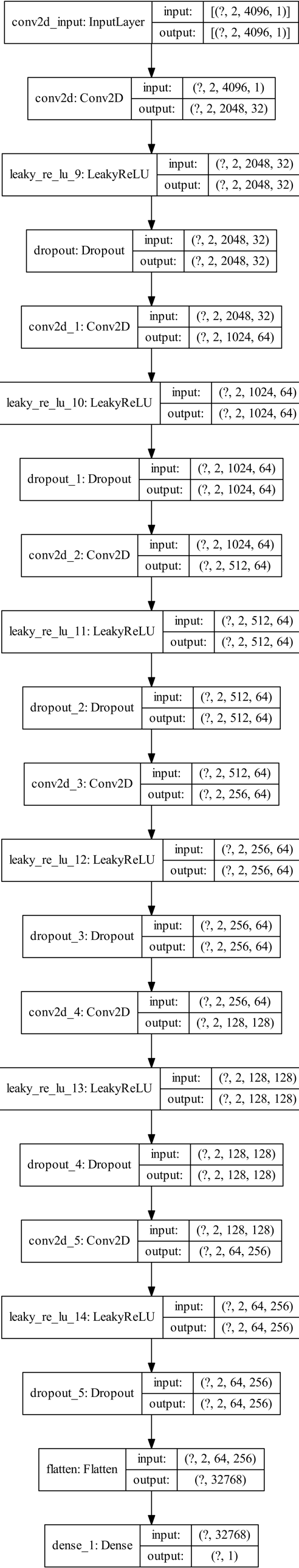

Supplement: S3 Appendix — (PDF) [file pone.0267452.s003.pdf]
